# The Effect of Waste Plastics on the Ageing Phenomenon of Bituminous Binders and Asphalt Mixtures

**DOI:** 10.3390/ma14206176

**Published:** 2021-10-18

**Authors:** Finn Hall, Greg White

**Affiliations:** School of Science, Technology and Engineering, University of the Sunshine Coast, Sippy Downs, QLD 4556, Australia; Fah003@student.usc.edu.au

**Keywords:** asphalt, binder, modification, recycled, plastic, polymer, ageing

## Abstract

The push for environmental sustainability in the civil engineering industry has resulted in an increased interest in the use of recycled construction materials, with one example being the use of waste plastic for the modification of bituminous binder in asphalt mixtures. Existing research has associated waste plastics with various binder and asphalt mixture performance enhancing properties. However, there is a lack of research on the age-related durability of waste plastic-modified roads. This research compared the effect of commercially available waste plastic binder modifiers on the ageing phenomenon of bituminous binders and asphalt mixtures, to the effect of conventional polymers SBS and EVA, through artificial bituminous binder and asphalt mixture ageing performed in a laboratory. The addition of polymers (both waste and virgin) resulted in an increase in binder stiffness after short-term ageing as the polymer content increased. The effect of the waste plastic on ageing was comparable to the effects associated with the conventional polymers, and it was concluded that the waste plastic binder modified products should be considered sustainable alternatives to standard polymers for bituminous binder and asphalt mixture modification.

## 1. Introduction

In 1960, the global production of plastic was roughly 7000 tonnes per year. In 2015, this figure reached 300 million tonnes [1]. The significant increase in production of plastic products does not come without a series of challenges. Because plastics are manufactured to be durable, most will not biodegrade, rather persisting in the environment for decades, centuries and even millennia. The severity of plastic pollution has resulted in experts describing it as a major global concern [2]. As a result, developments in civil engineering favouring environmentally friendly and sustainable infrastructure have seen an increase in popularity. One application of sustainable infrastructure is the development of sustainable pavements. In conventional road construction, man-made material is laid down to cover virgin ground, a significant amount of natural resource is used, non-renewable energy is consumed, the waste generated is sent to the landfill and different types of chemical gases are emitted to the environment [3].

The need for sustainable alternatives is evident and recycling secondary materials in asphalt mixtures has been identified as an example of sustainable pavements, as they reduce carbon emissions and promote environmental sustainability [4]. A promising development in sustainable pavements is the incorporation of waste plastic as a recycled material to modify the bituminous binder portion of an asphalt mixture, which initially appears to directly combat plastic pollution. Mashaan et al. [5] suggested that the use of waste plastic in asphalt mixtures aids in attaining environmental sustainability whilst promoting industrial–economic benefits. There currently exists significant research demonstrating the improved mechanical or engineering properties of asphalt mixtures modified with recycled plastic [6]. Some research has even found the effect of recycled plastic modification to be comparable to that associated with the conventional polymers, known as SBS and EVA [7]. However, one significant issue that remains unclear is the relative environmental ageing of asphalt mixtures modified with recycled plastic, compared to those modified with conventional polymers.

The aim of this research was to compare the accelerated laboratory ageing of bituminous binders and asphalt mixtures modified with two commercially available recycled plastic products to the ageing of an unmodified binder, as well as products modified with conventional polymers, such as SBS and EVA. Testing included binder penetration and softening point, as well as mixture stiffness modulus before and after artificial ageing representing up to 15 years of asphalt mixture ageing in the field.

## 2. Background

### 2.1. Waste Plactis in Asphalt Mixtures

For waste plastic modification of asphalt mixtures to be considered a viable technology, the recycled materials must offer equal or better performance than the pavement surface, at no greater cost [8]. Because the cost of pavement resurfacing is periodic, typically incurred ever 10–15 years, the effect on the durability of life expectancy of the surface is important.

Waste plastic has generally been associated with an improvement in asphalt mixture performance properties. For example, PET has been associated with an improvement in the deformation resistance and fatigue resistance of asphalt mixtures [9]. These findings are consistent with the findings of Dalhat and Wahhub [10], who shredded and ground low- and high-density polyethylene, as well as polypropylene, and wet mixed the recycled plastic products into unmodified bitumen prior to asphalt mixture manufacturing in the laboratory.

Separately, Naghawi et al. [11] evaluated the use of plastic waste as a low-cost asphalt binder modifier and found that pavement durability could increase in terms of resistance to fatigue cracking and rutting. Waste PET-modified asphalt mixtures have also resulted in similar performance enhancements to those previously specified, and Abdo [12] supports the use of waste plastics in asphalt mixture modification.

In the UK, three binders and mix modifiers have been commercially developed from waste plastics, with the aim of combatting plastic pollution, whilst improving the road network in the UK. This study focuses on the commercially available products known as MR6 (intended to be plastomeric) and MR10 (intended to be elastomeric) for which there currently exists extensive research.

White and Reid [8] demonstrated an increase in asphalt mixture modulus of 120–150% when MR6 was used as a bituminous binder modifier, added at 6% of the weight of the binder. This is consistent with other publications, as White [6] observed a significant decrease in rut depth associated with MR6. This is further supported by White and Magee [13] who, in their study, associated MR6 with various performance enhancements, including a reduction in wheel track rutting and an increase in resilient modulus, which is indicative of pavement strength. The findings supported the use of MR6 to enhance asphalt mixture performance, and therefore supported the use of waste plastics as a secondary material in asphalt mixtures.

In a more recent study focusing on the practicalities of using MR6 and MR10, White and Hall [14] recently concluded that the performance of these waste plastic modifiers is not necessarily affected by the mixing method used to incorporate the polymer. That is, the modified binder and modified mixture properties were not significantly different regardless of whether the MR6 and MR10 were wet mixed into the bituminous binder, or dry mixed into the asphalt mixture.

Despite the current publications focusing on the mechanical or physical properties of mixtures modified with MR6 and MR10, there is currently no research on the effect of these waste plastic products on the ageing phenomenon of bituminous binders and asphalt mixtures. This paper evaluates the effect of MR6 and MR10 on the ageing process of asphalt mixtures, compared to that associated with conventional polymers SBS and EVA.

### 2.2. Ageing of Bituminous Binders and Asphalt Mixtures

As with all products, asphalt mixtures succumb to inevitable ageing, leading to a variety of structural faults including fatigue cracking and surface ravelling [15]. In England alone, the average estimated cost of road repair was £17.2 million in 2018, with a further £19.5 million spent on compensation to drivers as a result of car damage due to structural faults in roads [16]. Any delay in the ageing process could reduce the number of faults in roads, ultimately reducing the need for regular repair work. If an additive (waste plastic or otherwise) slows the ageing process, this reduces the repair, resurfacing and compensation costs, and this would be welcomed by governments and would support the widespread use of these additives.

Indeed, the ageing phenomenon in asphalt mixtures has been described as one of the most important factors affecting its service life [17], and it impacts the retention of large aggregate particles via the adhesive characteristics of the binder with the aggregate particles [18]. The bituminous binder in the asphalt mixture is predominantly affected by environmental ageing [19]. Binder ageing occurs during the production of asphalt mixtures (short-term ageing) and while in service, due to exposure to the surrounding environment (long-term ageing) [20].

During short-term ageing, high temperatures during the processes of mixing, storage, spreading and compaction result in volatilisation. Volatilisation results in the evaporation of lighter constituents of binder, which results in short-term ageing [21]. Short-term ageing occurs at a very fast rate and results in a significant change in rheological properties of the binder, such as an increased viscosity and an increased stiffness [20]. Long-term ageing is the result of external conditions, such as temperature, traffic load and environmental effects, and results in a loss of road durability [18].

Asphalt mixture and binder ageing is routinely accelerated in laboratory conditions, allowing for controlled and rapid research on the effect of the ageing phenomenon. The Rolling Thin Film Oven Test (RTFO) is commonly used in the laboratory to simulate short-term binder ageing, likely to occur during the production and transportation of the binder. Long-term binder ageing is often artificially simulated through the use of a Pressurised Ageing Vessel (PAV). This test method intends to mimic field ageing, with one PAV cycle correlating to 10 years of in-field ageing.

In contrast, asphalt mixture samples are artificially aged through hot mixing in the laboratory to represent short-term ageing and are subject to PAV cycles to represent long-term ageing. For long-term asphalt mixture ageing, one PAV cycle represents 5 years of field ageing (Table 1).

Although the methods of laboratory asphalt mixture and bituminous binder ageing are widely used and are indicative of ageing likely to occur in the field, some have argued for more extensive test protocols. For example, most current laboratory ageing protocols do not reflect temperature gradients or cycles known to exist in real pavement surfaces, nor do they consider the effect of ultra-violet radiation or moisture on asphalt mixtures [22]. As such, a more realistic artificial ageing protocol is required [23]. Development of a new ageing simulation procedure, accounting for different environmental conditions, is desirable [20]; however, for the purpose of this research, the current best-practice test methods were used.

Various factors affect the rate of ageing of the binder, and mixing temperature and storage time during short-term ageing can influence the rate of ageing that occurs. In long-term ageing, temperature, exposure to UV and rainfall can influence the rate of binder ageing [22]. In addition to the factors induced during short-term and long-term ageing, it has been argued that mixture properties, for example, air void content, binder content, source and type of binder and the aggregate gradation can influence binder ageing by affecting the degree of exposure of the binder film to the environment [20].

### 2.3. Effect of Polymer Modification on Ageing

In a study evaluating the ageing properties of polymer modified asphalt mixtures, it was suggested that the degree of ageing is highly dependent on the polymer type and the level of polymer modification [24]. Reese and Predoehl [25] observed an improved resistance to ageing in polymer modified mixtures after a two-year service life in California; however, they indicated that further investigations were required to reach a stronger conclusion. Other reports have suggested that there is a significant degree of difficulty associated with quantifying the effects of polymer modification on asphalt mixture ageing, due to the effects of polymer degradation influencing ageing. Despite this, it has also been argued that SBS and crumb rubber modified binders offer a more stable response against the effects of ageing at medium–high temperatures [17]. This may only refer to specific ageing mechanisms; however, Wei et al. [19] concluded that unmodified bitumen and SBS modified binder have the same ageing behaviour when exposed to UV light.

In waste plastic asphalt mixture modification, one study associated waste PET additives with a greater ability to resist short-term ageing during construction. Results of laboratory long-term ageing research also suggest that PET modified asphalt mixtures have better fatigue resistance and longer fatigue life compared to an unmodified bitumen, and thus more durability in the longer term [5]. Despite this, there is a need for further research on the effects of waste plastics on the ageing of asphalt mixtures. This paper aims to compare the effect of the commercial waste plastic modifier products MR6 and MR10 on the ageing process of an asphalt mixture to the effects associated with conventional polymers SBS and EVA.

## 3. Materials and Methods

Asphalt mixtures were produced with binders modified with the commercial waste plastic products MR6 and MR10 at 6% weight of the binder, and with conventional polymer modified binder SBS and EVA at 2%, 4% and 6% of the weight of the binder, as well as unmodified penetration grade bitumen. The mixtures were otherwise nominally identical and reflected a common 10-mm-sized dense graded and Marshall designed mixture for road surfacing in the United Kingdom, meeting the requirements of BS EN 13108-1 (Table 2). Polymer-modified binders were blended in laboratory conditions using a 100–150 penetration-grade bitumen meeting the requirements of EN 12591. The asphalt mixtures were artificially aged in a laboratory oven and tested for stiffness modulus before and after ageing, to compare the extent of ageing. Modified binder samples were aged and tested for penetration and softening point to compare the effect of the different polymers on binder ageing. Both binder and mixture sample testing were performed in triplicate and the results were averaged. Control asphalt mixture samples were produced with 50–70 penetration-grade bitumen meeting the requirements of EN 12591. The recycled plastic modified samples all included 6% (by mass of the unmodified bitumen) of the commercial products MR6 or MR10. The SBS and EVA modified binders were produced with 2%, 4% and 6% polymer content, by mass of the unmodified bitumen.

### 3.1. Binder Ageing

Both modified and unmodified binder samples were subject to artificial laboratory aging via the Rolling Thin Film Oven test (RTFO) to simulate short-term ageing of the binder, likely to occur during mixing, transport and paving. For the RTFO test, 35 g of binder was weighed out and placed in glass bottles, which were then placed inside a rotatory oven at 163 °C for 85 min (BS EN 12607-1). RTFO ageing is widely used and gives a relatively sufficient indication of short-term binder ageing in practise. The binder samples were tested for penetration and softening point in accordance with BS EN 1426-1427, before and after short-term laboratory ageing through the RTFO test.

After short-term ageing through the RTFO test, the remaining binder samples were subject to further ageing in a Pressurised Ageing Vessel (PAV) to simulate long-term binder ageing in accordance with BS EN 14769. A 50 g sample of post RTFO binder was placed on a circular tray and the tray was placed inside the pressurised ageing vessel, which exposed the samples to pressurised air at 2.1 MPa at a temperature of 110 °C for 20 h. Once removed from the PAV, the aged samples were tested for penetration and softening point (BS EN 1426-1427) to provide a comparison between short-term and long-term ageing.

### 3.2. Asphalt Mixture Ageing

Bulk asphalt mixture samples were manufactured in a laboratory mixer using the modified binders specified, as well as an unmodified 100–150 pen binder. The bulk samples were compacted in a gyratory compactor to a target air void content of 6%, prior to artificial ageing via an oven. The samples were placed in the oven at a temperature of 135 °C for 72 h, and then removed and tested for stiffness modulus at 20 °C, in accordance with BS EN 12697-26, which is an indirect tensile test. All testing was performed on triplicate specimens to obtain a mean value. The asphalt mixture samples were then placed back in the oven, repeating the ageing cycle, totalling up to 216 h of ageing, with the stiffness modulus measured after every 72-h cycle. The difference in stiffness modulus after each cycle represented the extent of ageing of the asphalt mixture samples.

## 4. Results

The reduction in penetration and the increase in softening point were used as indicators of binder ageing. Table 3 summarises the triplicate results obtained for binder penetration. Table 4 summarises the softening point results, including the unaged, post RTFO treatment and post PAV treatment. For the asphalt mixtures, the increase in stiffness modulus was used as the indicator of ageing. Table 5 summarises the asphalt mixture stiffness modulus, unaged and after every ageing cycle.

## 5. Discussion

### 5.1. Binder Ageing

**Figure 1:**
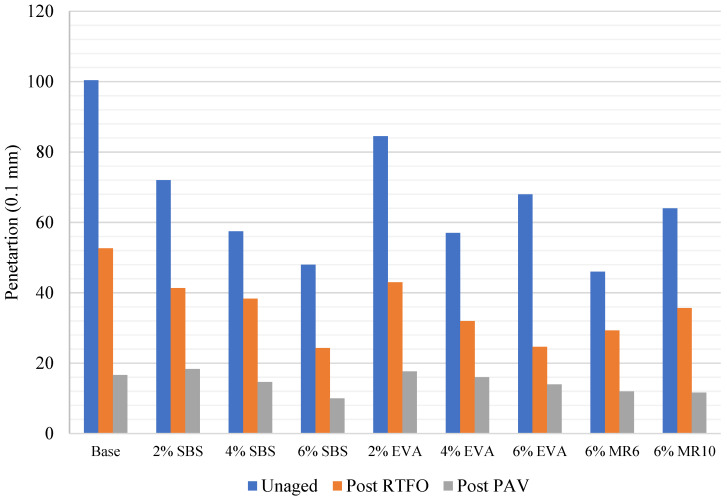
Binder average penetration before and after ageing.

**Figure 2:**
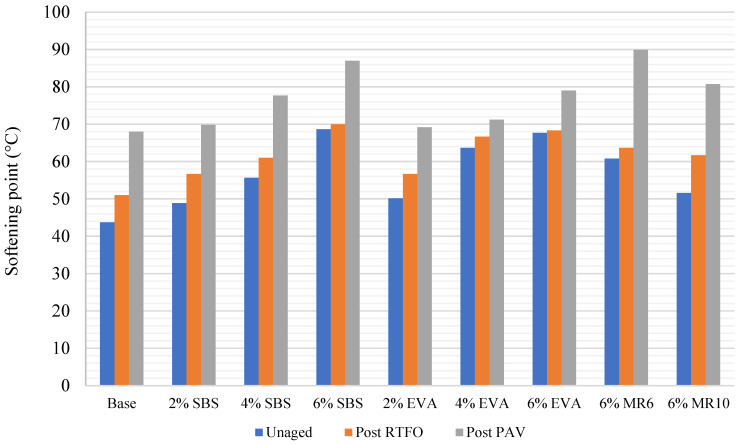
Binder average softening point before and after ageing.

Artificial ageing of binder samples confirmed that the ageing process affects the physical characteristics of the binder. Every binder sample saw a significant decrease in penetration as a result of short-term ageing via the RTFO (Figure 1), as well as an increase in softening point (Figure 2). A further decrease in penetration was observed in all samples after the long-term binder ageing via the PAV (Figure 1), as well as a further increase in softening point (Figure 2).

The decrease in penetration and increase in softening point observed as a result of short-term binder ageing through the RTFO were consistent with previous research [26] and reflect a hardening of the binder through an increase in binder viscosity as a result of oxidation in the short-term ageing phase. The decrease in penetration reflects an increase in binder stiffness, which is a result of a loss of volatile oils during short-term ageing. The softening point test results also indicate that the binder had hardened, as every sample saw an increase in softening point post RTFO (Figure 2). This may reflect volatilisation, where oil-like compounds were evaporated from the mixture. On average, the penetration results reduced by 46% after RTFO treatment and by 78% after both the RTFO and PAV treatment. At the same time, the average softening point result increased by 10% after RTFO treatment and by 37% after both the RTFO and PAV treatments. The results obtained from binder ageing support previous claims that, as binder ageing occurs in both the short-term and long-term, the binder will become more viscous.

### 5.2. Mixture Ageing

**Figure 3:**
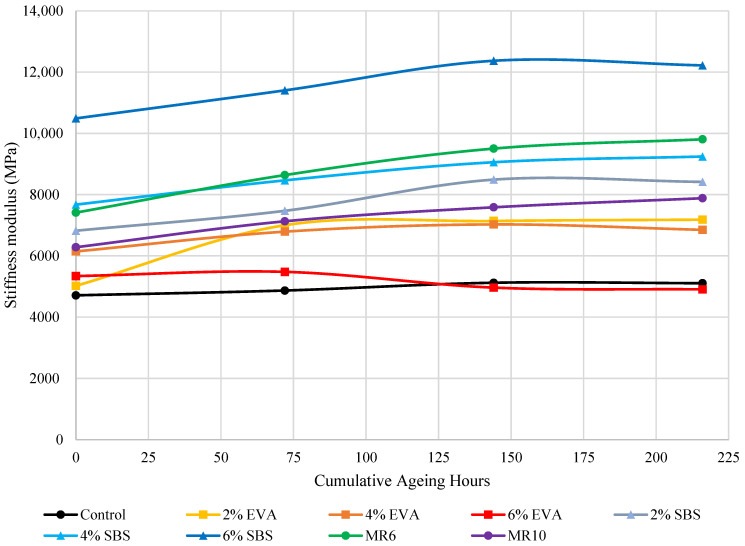
Asphalt mixture stiffness modulus before and after ageing cycles.

**Figure 4:**
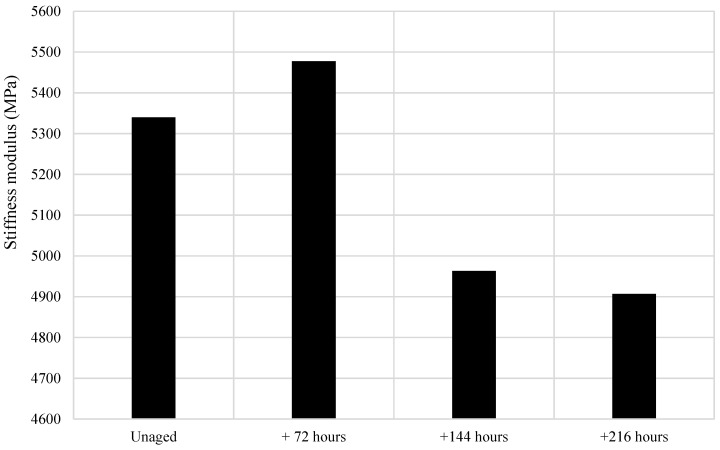
Average 6% EVA asphalt mixture stiffness modulus before and after ageing.

After the first ageing cycle of 72 h, every mixture saw an increase in stiffness modulus, followed by a further increase after the second ageing cycle and a slight increase after the third cycle. (Figure 3). Generally, as the mixtures aged, the stiffness modulus increased, with the exception of 6% EVA, which was associated with a significant decrease in stiffness modulus after the second ageing cycle (Figure 4). On average, the asphalt mixture stiffness modulus increased by 13% after one ageing cycle, by 19% after two ageing cycles and by 23% after three ageing cycles. That is, the average asphalt mixture modulus continued to increase with artificial ageing cycles, but at a reducing rate.

The increase in asphalt mixture stiffness modulus after each ageing cycle reflects the increase in bituminous binder rigidity. The increase in stiffness modulus observed after ageing is consistent with previous publications and supports similar findings that, as the stiffness modulus increases due to ageing, the mixture is likely to become excessively hard and brittle, thus making it susceptible to disintegration and fatigue cracking at low temperatures [20]. The flexibility of the pavement reduces as ageing occurs, due to the significant increase in stiffness modulus. However, an increase in stiffness and cohesion of an asphalt mixture would likely result in an improved resistance to permanent deformation and load bearing capacity, meaning mixture property changes as a result of ageing are not necessarily negative, although they are likely to be indicators of reduced surface life expectancy, particularly in local road applications where environmental ageing usually triggers resurfacing.

### 5.3. Effect of Polymer Type on Binder Ageing

The binder that saw the greatest change in penetration after short-term ageing (RTFO) was 6% EVA, with a 66% decrease (Figure 1). However, the significant percentage change in penetration reflects the high penetration exhibited in the unaged 6% EVA binder, which was higher than the penetration of unaged 4% EVA. Typically, as polymer content increases, the penetration increases as the binder becomes stiffer [6], and so the 6% EVA binder results appear to be anomalous. Removing the 6% EVA binder results, the binder that saw the greatest change in penetration after short-term ageing was 2% EVA, with a 49% change (Figure 1). Conversely, 4% SBS saw the smallest change in penetration as a result of short-term ageing, with a 34% decrease (Figure 1). This is consistent with previous research, as it has been previously highlighted that SBS-modified binders are less susceptible to ageing than unmodified bitumen [27]. Previous research has recognised that, after oxidation induced ageing, the hardening of EVA modified binders was more pronounced than in binders modified with SBS, on the basis that SBS-modified binder ageing causes both polymer degradation and bituminous binder oxidation, while EVA modification induces oxidative hardening of the materials [17].

The penetration of the MR6- and MR10-modified binders responded similarly to EVA and SBS short-term ageing, with a 37% and a 44% decrease, respectively. After long-term ageing, MR10 saw the greatest decrease in penetration, followed by the 4% SBS (Figure 1). This was consistent with the softening point results post-PAV, with the softening point of MR6 decreasing by 40% after long-term ageing, which was the greatest decrease amongst the mixtures. MR10 saw a decrease in softening point of 31% after long-term ageing. The mixture had the smallest change in softening point after artificial long-term ageing was 4% EVA (Figure 2). That is, the EVA-modified binder was more affected by short-term ageing, but was subsequently less affected by long-term ageing.

### 5.4. Effect of Polymer Type on Mixture Ageing

The 2% EVA-modified asphalt mixture was associated with the greatest change in stiffness modulus as a result of ageing, with a 40% increase after the first ageing cycle, a 42% increase after the second and a 43% increase after the third. The control mixture saw the smallest change in stiffness modulus after each ageing cycle, and the MR6 and MR10 mixtures were comparable, with a change in modulus of 14% and 17%, respectively, after one ageing cycle, and a change of 20% to 30% after the second and third cycle (Figure 3).

### 5.5. Behaviour of the 6% EVA Asphalt Mixture

The stiffness modulus of the 6% EVA mixture increased after one ageing cycle, but saw a decrease after the second ageing cycle and a further decrease after the third cycle (Figure 4). This trend was anomalous, as every other asphalt mixture saw an increase in stiffness modulus as the asphalt mixture aged, which supports previous research suggesting that stiffness modulus conventionally increases as asphalt mixture ages.

The 6% EVA decrease in stiffness modulus indicates cracking of the asphalt core, subsequently highlighting that the asphalt mixture samples failed. The asphalt mixture failure would likely be a result of binder ageing, as the aggregate has less influence on the extent of ageing. The results of binder ageing on the 6% EVA sample indicate that, after short term ageing, the binder saw the greatest decrease in softening point (Figure 2), which suggests that EVA-modified binders may be more susceptible to ageing in the short-term compared to SBS and the waste plastic products MR6 and MR10. Indeed, if a binder is more susceptible to ageing, the asphalt mixture is more likely to experience failures, which was observed in the 6% EVA sample. However, as stated previously, the penetration of the unaged 6% EVA binder was higher than the penetration of the 4% EVA binder, which is considered an anomaly, and so the anomalous 6% EVA mixture result may relate to the anomalous 6% EVA binder results.

As previously discussed, short-term binder ageing occurs due to oxidation, and the effect of the oxidative process is polymer dependent. Therefore, the extent of ageing depends on the polymer type [18]. In a study evaluating the effect of ageing on EVA- and SBS-modified binders, it was observed that, at 2% EVA content, the polymer showed signs of low compatibility with the unmodified bitumen. It was concluded that, in the case of EVA-modified binder, although co-existing, the polymer and bitumen remain separated without forming a network. The lack of network correlates to the response of the modified binder, in which the EVA behaves only as a reinforcement without increasing the elasticity of the bituminous binder [18]. This may offer some explanation as to why the 6% EVA core failed; however, the complexity of the chemical interactions taking place between the polymer and bitumen introduces a level of difficulty in reaching a conclusion as to why this only occurred in the EVA mixture beyond the scope of this research.

### 5.6. Effect of Polymer Content on Binder Ageing

**Figure 5:**
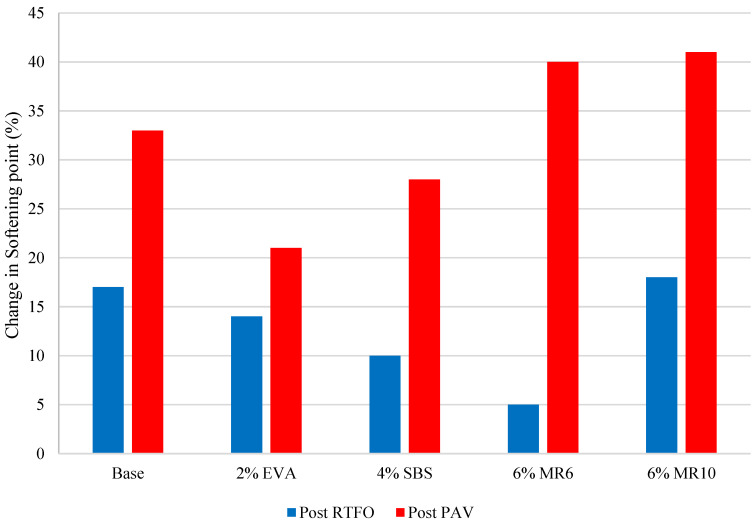
Change in average binder softening point before and after ageing treatments.

**Figure 6:**
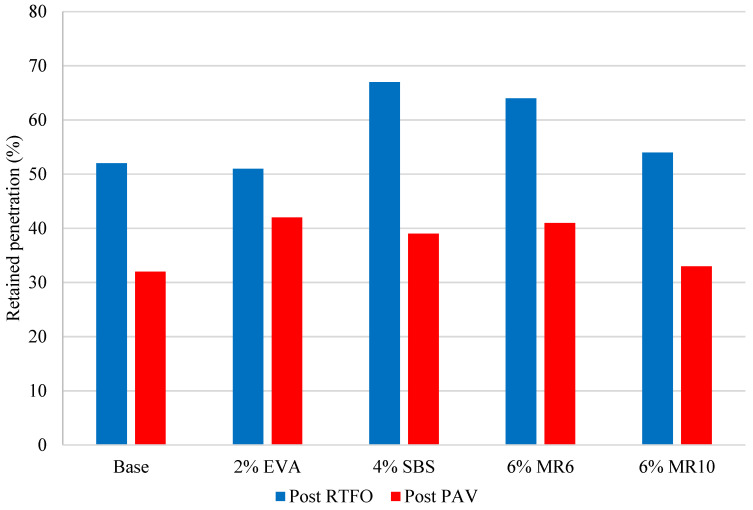
Retained average binder penetration before and after ageing treatments.

The waste plastic binder modifier products evaluated as part of this research have previously been compared in terms of performance to the conventional polymers EVA and SBS [28]. It was concluded that MR6 and MR10, at a dosage of 6% of the binder volume, had a Marshall stability comparable to 2% to 3% conventional polymer, and comparable deformation resistance to 4% to 6% conventional polymer [29]. Separately, based on an internal industry review relating to PMB use in Europe, the typical polymer content for SBS is 4% by weight of the binder, and the typical polymer content for EVA is 2% by weight of the binder [30]. For this reason, and based on the performance comparison, conventional polymer contents of 2% EVA, 4% SBS and 6% MR6 and MR10 were compared. From the binders selected for comparison, MR10 was associated with the greatest change in softening point after short-term ageing (Figure 5). The binder with the greatest change in penetration after short-term ageing was 2% EVA (Figure 6). The change in binder properties of the MR6-modified binder and the 4% SBS binder were comparable after short-term ageing. After long-term ageing, both waste plastic-modified binders exhibited an increase in softening point of approximately 40%, which is significantly higher than the increase associated with SBS and EVA. The retained penetration, however, was consistent for all four modified binders after long-term ageing (Figure 6).

### 5.7. Effect of Polymer Content on Mixture Ageing

**Figure 7:**
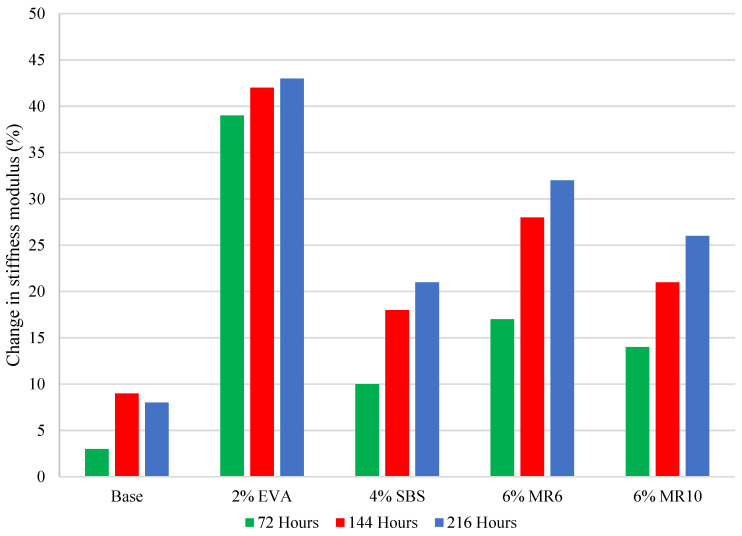
Average increase in mixture stiffness modulus after ageing cycles.

Similarly, asphalt mixtures modified with 2% EVA, 4% SBS and 6% MR6 and MR10 were compared to determine the effect of polymers on asphalt mixture ageing. After the first ageing cycle, MR10 saw a similar change in stiffness modulus to 4% SBS, with a relatively small change of 10% to 20%, compared to 2% EVA which saw a significantly greater (38%) increase than the other three mixtures (Figure 7). The change in stiffness modulus of MR10 and SBS remained relatively consistent after each ageing cycle, suggesting that the effect of MR10 and SBS, added at a standard dosage, on the ageing process of asphalt mixtures is comparable. One possible explanation for this is that, during binder ageing, SBS undergoes polymer degradation, which may reduce the stiffening of the asphalt mixture, observed through the consistent and relatively small change in stiffness modulus. The change in stiffness modulus for the MR6 mixture was greater than that of the SBS mixture and smaller than the EVA mixture after each ageing cycle. The comparable asphalt mixture ageing between MR10 and SBS may reflect the more elastomeric nature of the MR10 waste plastic product [6].

### 5.8. Comparison of Trends in Binder and Mixture Results

The binder that saw the smallest change in penetration after short-term artificial ageing was 2% EVA, indicating a stiffer binder. The asphalt mixture that saw the greatest increase in stiffness modulus after each ageing cycle was also the 2% EVA, which supports the claim that binder ageing and asphalt mixture ageing are correlated, and that both test methods are reliable indicators of expected in-field ageing. Similarly, the binder sample least affected by the ageing process was 4% SBS, which saw the smallest change in properties after short-term ageing. The 4% SBS asphalt mixture showed a significantly smaller change in stiffness modulus, which reflects the properties of the modified binder. Consequently, the relative ageing of asphalt mixtures modified with waste plastic, compared to those with conventional SBS- and EVA-modified binder, was generally consistent regardless of whether the binder properties or the mixture properties were focused on.

## 6. Conclusions

The addition of SBS and EVA polymers (both virgin and waste) resulted in a stiffer binder after short-term ageing, observed through a greater decrease in penetration as polymer content increased. SBS and the waste plastic products MR6 and MR10 added at a standard (6% of bitumen weight) dosage resulted in a similar change in binder properties after short-term binder ageing. After long-term binder ageing, the waste plastic products exhibited a greater increase in softening points than SBS and EVA; however, the retained penetration was considered comparable for all modified binders. Ageing of polymer modified asphalt mixture samples resulted in an increase in stiffness modulus after each ageing cycle, reflecting an increase in hardness and, subsequently, a decrease in flexibility. The effect of ageing was dependent on the type of polymer and polymer content, with a higher polymer content resulting in a generally stiffer binder. At 6% (by binder mass), MR10 resulted in a change in stiffness modulus comparable to that associated with conventional polymer SBS after each asphalt mixture ageing cycle. This likely highlights the elastomeric properties of MR10. The change in properties due to ageing can be considered comparable between waste plastic products MR6 and MR10, and conventional polymers SBS and EVA, at their respective typical dosage rates. However, the asphalt mixture stiffness modulus and bituminous binder properties measured in this research are not direct indicators of field ageing, and rheological and/or chemical properties should be considered in future research.

## Figures and Tables

**Table 1:** Accelerated ageing protocols.

Sample Type	Field Condition Being Represented by Laboratory Treatment			
	Produced and Paved	5 Years Field Aged	10 Years Field Aged	15 Years Field Aged
Binder	One RTFO cycle	-	One PAV cycle	-
Mixture	Hot mixed in the laboratory	One PAV cycle	Two PAV cycles	Three PAV cycles

**Table 2:** Asphalt mixture properties.

**Property**	**Test Method**	**Limits**	**Target**
Binder content (by mass) (%)	BS EN 12697-1	4.5–5.5	5.2
Voids in the aggregate (%)	BS EN 12697-8	18.0–22.0	20.0
**Percentage Passing (%) Standard Sieve Size (mm)**			
**Sieve Size (mm)**	**Test Method**	**Limits**	**Target**
10	BS EN 12697-2	90–100	99
6.3		62–68	68
2		25–33	32
1		17–26	22
0.063		4–8	7

**Table 3:** Binder penetration results.

Material	Penetration (0.1 mm)		
	Unaged	Post RTFO	Post PAV
Control	93	52	17
	106	51	17
	103	55	16
2% SBS	68	41	20
	76	41	19
	72	42	16
4% SBS	51	38	14
	64	39	15
	58	38	15
6% SBS	48	24	10
	48	23	10
	48	26	10
2% EVA	78	43	17
	91	43	20
	85	43	16
4% EVA	57	31	15
	57	33	17
	57	32	16
6% EVA	68	22	12
	68	26	15
	68	26	15
MR6	46	30	10
	46	29	14
	46	29	12
MR10	64	33	12
	64	39	11
	64	35	12

**Table 4:** Binder softening point results.

Material	Softening Point (°C)		
	Unaged	Post RTFO	Post PAV
Control	43	52	68
	44	50	67
	44	51	68
2% SBS	48	57	70
	49	56	70
	49	57	70
4% SBS	57	61	78
	54	61	77
	56	61	78
6% SBS	69	69	87
	69	70	87
	68	71	89
2% EVA	50	57	70
	51	57	69
	50	56	69
4% EVA	64	67	70
	64	66	71
	63	67	72
6% EVA	68	67	79
	69	69	78
	66	69	79
MR6	61	64	89
	61	63	90
	60	64	90
MR10	52	62	81
	50	61	81
	50	62	82

**Table 5:** Asphalt mixture stiffness modulus results.

Material	Stiffness Modulus (MPa) after Cycles (Hours) of Ageing			
	Unaged	72 h	144 h	216 h
Control	4740	4845	5199	5350
	4630	4839	4963	5080
	4760	4920	5205	4892
2% SBS	6950	7013	8470	8150
	7080	7375	8400	8360
	6430	8026	8600	8740
4% SBS	7300	7836	8850	9300
	7180	7578	8190	8390
	8540	9981	10,130	10,040
6% SBS	11,580	12,744	13,350	12,850
	10,080	10,235	11,300	11,058
	9810	11,235	12,460	12,750
2% EVA	5030	6807	7520	7810
	5130	6882	6910	7100
	4910	7318	6990	6640
4% EVA	6580	6951	6830	6410
	6150	6657	7100	6900
	5710	6767	7150	7240
6% EVA	5220	4801	4300	3950
	4570	5880	5550	5570
	6230	5751	5040	5200
MR6	7150	7646	8290	8750
	7330	9228	9540	10,480
	7760	9041	10,680	10,189
MR10	6120	7047	7320	7640
	6130	6357	6830	7210
	6600	7985	8610	8800

## Data Availability

The data presented in this study are available on request from the corresponding author.
